# Neural substrates related to memory consolidation of learning multiple motor sequences during wakeful rest

**DOI:** 10.1016/j.isci.2026.117653

**Published:** 2026-10-26

**Authors:** Seojin Yoon, Antoine Caraballo, Sungshin Kim

**Affiliations:** 1 Department of Healthcare Digital Engineering, Hanyang University, Seoul, Republic of Korea; 2 Department of Data Science, Hanyang University, Seoul, Republic of Korea; 3 Department of Artificial Intelligence, Hanyang University, Seoul, Republic of Korea; 4 Center for Neuroscience Imaging Research, Institute for Basic Science, Suwon, Republic of Korea

**Keywords:** motor sequence learning, memory consolidation, hippocampus, MRI

## Abstract

Motor learning has long been considered independent of the hippocampus, but recent evidence suggests that the hippocampus contributes to motor memory consolidation during wakeful rest. However, whether hippocampal reactivation supports consolidation during interleaved rest remains unclear. We investigated the fMRI evidence of motor memory consolidation of learning multiple motor sequences during wakeful rest. We found that while sensorimotor regions exhibited sequence-specific activity patterns during practice, these were not reliably discriminable in the sensorimotor regions and hippocampus during interleaved rest. In contrast, neural activity patterns in the right hippocampus, right putamen, and sensorimotor regions during interleaved rest showed greater similarity to post-learning rest patterns than pre-learning rest patterns. These findings suggest that under highly interfering, multi-sequence learning conditions, the consolidation of motor memories may not be related to a specific sequence but may instead reflect a generalized or abstract representation of sequences, potentially to stabilize and minimize interference among newly acquired skills.

## Highlights

• Motor sequence learning practice shows sequence-specific activity but not interleaved rest
• Interleaved rest activity becomes more similar to post-learning rest
• Consolidation under high interference may rely on generalized representations

## Graphical abstract

**Figure:**
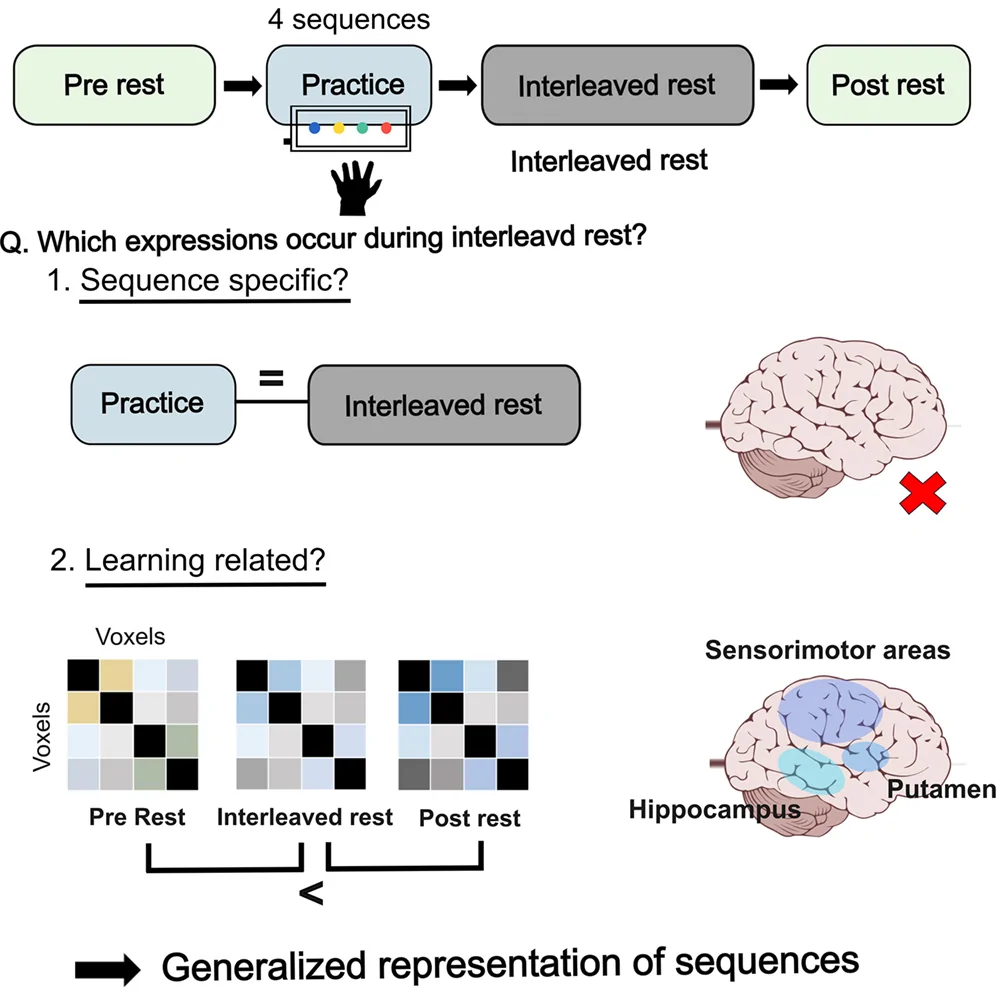

## Teaser

Natural sciences; Biological sciences; Neuroscience; Clinical neuroscience

## Introduction

Consolidation of motor memory enhances performance by transforming initial fragile memories into more stable ones after the completion of training.^1^ The offline performance gain due to consolidation may occur between practice sessions with a sufficiently long break as well as overnight.^2^^,^^3^ Neuroimaging studies have suggested that sensorimotor regions, such as the primary motor cortex, striatum, and cerebellum, underlie motor memory consolidation,^4^^,^^5^^,^^6^ specifically through cortico-striatal networks that contribute to sequence learning and cortico-cerebellar networks that support motor adaptation.^6^ Beyond the sensorimotor regions, recent studies suggest that the hippocampus, traditionally considered independent of motor memories, may play a role in motor sequence learning (MSL). Evidence of its important role in consolidating motor memory during sleep comes from both animal studies^7^ and human studies.^8^^,^^9^^,^^10^^,^^11^ The consolidation of motor memory appears to occur through the cortical-hippocampal interaction, as suggested in the complementary learning system (CLS) theory.^12^ Simultaneous human EEG-fMRI studies^9^^,^^10^ and a recent animal study^7^ demonstrated that the functional coupling between sharp-wave ripples (SWRs) in the hippocampus and slow oscillations in the motor cortex plays a key role in motor memory consolidation.

The hippocampus appears to be involved in motor memory consolidation during wakeful rest after practice^8^^,^^13^ and even during short rest periods interleaved with practice.^14^^,^^15^^,^^16^ Together, these studies suggest that memory consolidation due to hippocampal plasticity may occur at multiple time scales, ranging from seconds to days. Notably, the rapid consolidation in seconds may enhance short-term performance during interleaved rest periods, a phenomenon known as micro-offline gains (*MOGs*). Neural replay or reactivation in the hippocampus during rest periods has been suggested as a mechanism underlying the rapid consolidation of motor memories.^15^^,^^16^^,^^17^ Blood-oxygen-level-dependent (BOLD) signals in the hippocampus during interleaved rest were shown to correlate with *MOGs*, and functional changes were followed by rapid micro-structural changes of the order of minutes.^15^ In a magnetoencephalography (MEG) study, neural activity related to a practiced sequence appeared to be replayed in the sensorimotor cortices and hippocampus during interleaved rest periods, and the extent of replay correlated with *MOGs*.^16^ A study of amnesic patients with hippocampal damage demonstrated impaired *MOGs* compared with age-matched controls, supporting the necessity of the hippocampus for rapid motor memory consolidation.^18^ These studies have suggested that sequence-specific neural activity could be replayed in the hippocampus during offline periods, resulting in *MOGs*.

In this study, we used functional magnetic resonance imaging (fMRI) coupled to multivoxel-pattern analysis to investigate whether hippocampal BOLD patterns during interleaved rest reflect the sequence-specific reactivation of motor memories. We hypothesized that if the hippocampus supports rapid offline consolidation, the multivariate patterns associated with specific sequences during practice would be reinstated during the subsequent short rest period. Indeed, evidence from previous studies has shown that neural activity patterns in sensorimotor regions can be reliably distinguished across motor sequences and become more stable due to consolidation.^19^ However, it remains unclear how BOLD activity patterns during interleaved rest reflect the hypothesized replay of motor sequences in the hippocampus. Briefly, we designed a human fMRI experiment in which participants practiced multiple motor sequences in a pseudorandom order with interleaved rest periods. This randomized order was chosen specifically to prevent deterministic preparation for the upcoming sequence and minimize planning. Our primary aim was to analyze the similarity between sequence-specific BOLD patterns observed within the hippocampus during active practice periods and those during interleaved rest periods. Contrary to our expectations, while we found evidence of sequence-specific patterns during practice in the sensorimotor regions, we did not find any evidence of sequence-specific patterns within the hippocampus during interleaved rest or practice. This lack of sequence-specific patterns encouraged us to conduct an exploratory multi-voxel correlation structure (MVCS) analysis to investigate whether broader, learning-related representational structures in the hippocampus and sensorimotor regions, including the striatum, could extend beyond the entire practice session by comparing the interleaved rest with the pre- and post-learning resting state (pre- and post-RS). We found that the right posterior hippocampus and right putamen exhibited a persistence of patterns observed during interleaved rest into post-learning rest. As such, we developed a post hoc interpretation: under high-interference conditions, such as learning multiple sequences simultaneously, the brain may integrate information across sequences to form a generalized motor schema, an abstract representation of their shared structure.^20^^,^^21^

## Results

### Successful learning of four motor sequences

In the MRI scanner, 25 right-handed participants performed an MSL task^14^^,^^15^^,^^16^ with their left non-dominant hand, which consisted of a series of trials alternating between 10-s active practice periods and 20-s rest periods. Their performance, measured as the correct sequence typing speed, with higher values indicating better performance (see STAR Methods for details), improved significantly across blocks (*F* (23, 115) = 29.35, *p* < 0.001, *η*^2^ = 0.26) and differed significantly between sequences (*F* (3, 15) = 9.78, *p* < 0.001, *η*^2^ = 0.062; Figures 1B and S1). However, the interaction between blocks and sequences was not significant (*F* (69, 345) = 1.07, *p* = 0.28, *η*^2^ = 0.016), indicating that the rate of speed improvement remained consistent regardless of the sequence practiced.

**Figure 1:**
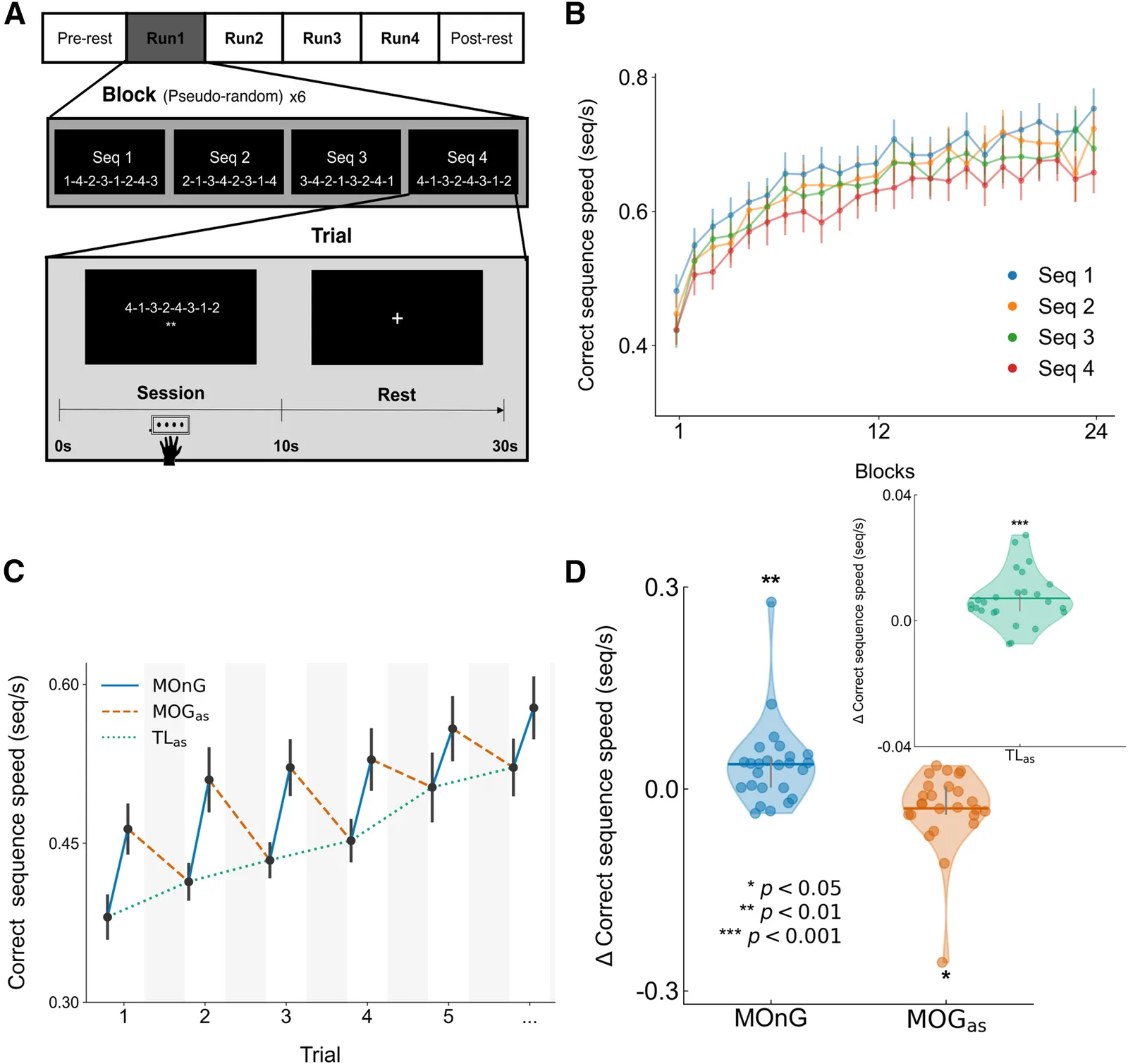
Experiment design and behavior performance (A) Participants performed a finger tapping task involving multiple motor sequences in the MRI scanner. Four different finger sequences were presented in a pseudorandom order within each block. Each sequence was displayed on the screen for a 10-s active practice period, followed by a 20-s rest period. An asterisk was displayed on the screen when the participant executed the full sequence. Multiple asterisks could appear on the screen. (B) Group results across participants of performance. Performance was measured by the speed of correct sequence completion (seq/s), where higher values indicate better performance. Data are represented as mean ± SEM. (C) Micro-online gains (*MOnGs*) are measured as the difference in correct sequence speed between the first and last correct sequence within a practice period. Micro-offline gains across sequences (*MOG*_*as*_) are measured as the difference between the last correct sequence speed in one practice period and the first correct sequence speed in the subsequent practice period. Total learning across sequences (*TL*_*as*_) is measured as the difference between the first correct sequence speed in one practice period and first correct sequence speed in the subsequent practice period. Data are represented as mean ± SEM (black). (D) Average *MOnG*, *MOG*_*as*_, and *TL*_*as*_ during the learning phase. Gains were calculated by averaging the performance improvements across all trials until each participant reached their asymptotic performance level (see the STAR Methods for details). Horizontal solid lines represent the mean, and the vertical gray lines span the 25th to 75th percentiles. Statistical significance was determined using a two-sided *t* test (∗*p* < 0.05, ∗∗*p* < 0.01, ∗∗∗*p* < 0.001).

Wakeful rest during the learning phase is thought to support MSL through rapid offline consolidation.^14^^,^^15^^,^^16^ Consistent with previous studies, we defined the learning phase as the period preceding the point at which participants reached 95% of their overall performance level. During this phase, we quantified “micro-online gains” (*MOnGs*), “micro-offline gains across sequences” (*MOG*_*as*_), and “total learning across sequences” (*TL*_*as*_). *MOnG* represents performance changes during a practice period calculated as the difference between first and last sequence execution within a trial. *MOG*_*as*_ represents performance changes during an interleaved rest period regardless of the specific sequence, calculated as performance difference between the last execution of a previous trial and the first sequence execution of the successive trial. *TL*_*as*_ represents the performance changes across both practice and interleaved rest periods, calculated as the difference between first execution of a trial and the first execution of the following trial (Figure 1C; see STAR Methods for details). For all three metrics, positive values indicate an improvement in performance (increased correct sequence speed), while negative values indicate a decline. Our results revealed significant *MOnG* (*T*(24) = 2.90, *p* < 0.01), demonstrating that participants improved their performance through the practice of four sequences (Figure 1D). Also, we observed a significant negative effect in *MOG*_*as*_ (*T*(24) = −2.50, *p* < 0.05). In contrast, *TL*_*as*_, which captures the cumulative learning effect, demonstrated a significant positive effect (*TL*_*as*_: *T*(24) = 2.50, *p* < 0.05). These effects remained significant when computed across all trials of the experiment (*MOnG*: *T*(24) = 2.56, *p* < 0.05; *MOG*_*as*_: *T*(24) = −2.35, *p* < 0.05; *TL*_*as*_: *T*(24) = 4.01, *p* < 0.001).

### Representation of motor sequences in sensorimotor regions

To identify sequence-specific activity patterns during encoding, we investigated BOLD responses associated with motor sequences during the practice periods. Using a searchlight approach with the crossnobis distance metric,^22^ we measured the representational dissimilarity between sequence pairs (Figure S2). This exploratory analysis revealed significant pattern dissimilarity across sensorimotor regions (Figure 2A), specifically in the bilateral premotor cortex, M1, and parietal lobule (uncorrected *p* < 0.05; Figure 2B).

**Figure 2:**
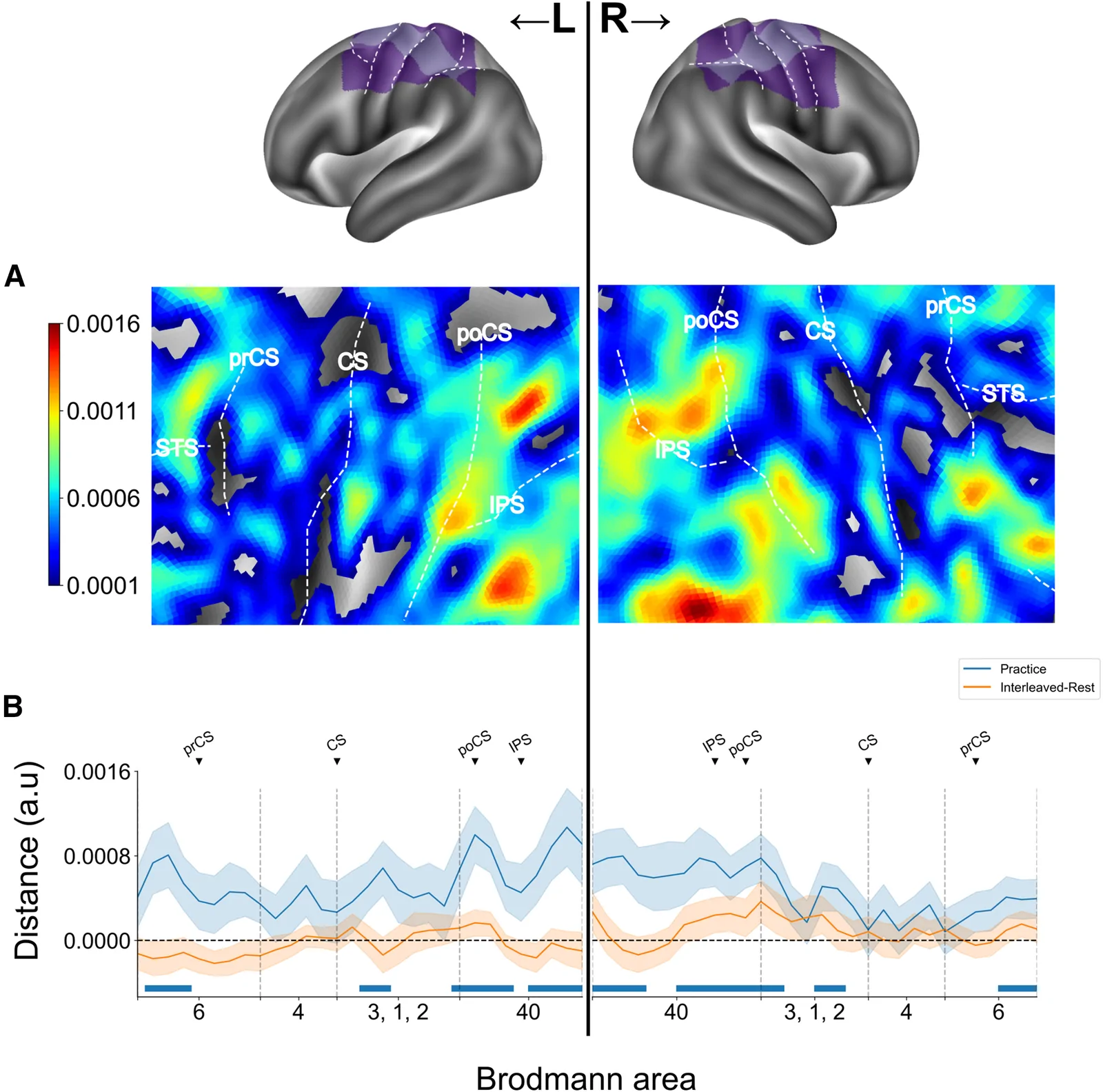
Mean dissimilarity within the sensorimotor areas during practice and interleaved rest periods (A) Surface visualization of the brain, highlighting the analyzed region in purple within a 3D representation. The group-averaged results show the mean crossnobis distances for sequence pairs during the practice period, obtained using the whole-brain searchlight approach. Anatomical landmarks are labeled as follows: CS (central sulcus), prCS (precentral sulcus), poCS (postcentral sulcus), STS (superior temporal sulcus), and IPS (intraparietal sulcus). (B) Profile ROI analysis along the strip indicated in light purple area of Figure 2A, showing the mean crossnobis distance (±SEM) during the practice and interleaved-rest periods. The rectangles above the *x* axis indicate significant regions (uncorrected *p* < 0.05, two-sided *t* test). Significant effects were observed in sensorimotor areas only during the practice period.

Next, we examined potential reactivation during the interleaved rest periods. Based on the premise that reactivation would increase the similarity between task and rest states, we computed a reactivation index that quantifies the reduction in dissimilarity between practice and its subsequent rest periods, where a positive index reflects greater similarity between task and rest (Figure S2). While an initial exploratory searchlight analysis identified significant positive clusters in various regions, we further verified whether these regions exhibited reliable sequence-specific representations (i.e., mean dissimilarity >0) within the interleaved rest period itself. Crucially, we observed minimal overlap between these reliable rest representations and the reactivation index, suggesting that the initial positive indices were likely not driven by robust sequence reactivation (Figure S4).

### Absence of hippocampal reactivation during interleaved rest periods

To investigate whether sequence-specific patterns were initially encoded during the task and subsequently reactivated, we assessed the mean dissimilarity of hippocampal BOLD activity slice-by-slice along the longitudinal axis during both the practice and interleaved rest periods (Figure S2). Overall, mean dissimilarity values in the hippocampus fluctuated around zero across both hemispheres (Figure 3). Although one isolated slice during the practice period and two isolated slices during the rest period exhibited a negative mean dissimilarity (two-sided *t* test, *p* > 0.05), this localized effect was not observed in adjacent slices. Furthermore, no slices yielded a positively significant reactivation index (Figure S4).

**Figure 3:**
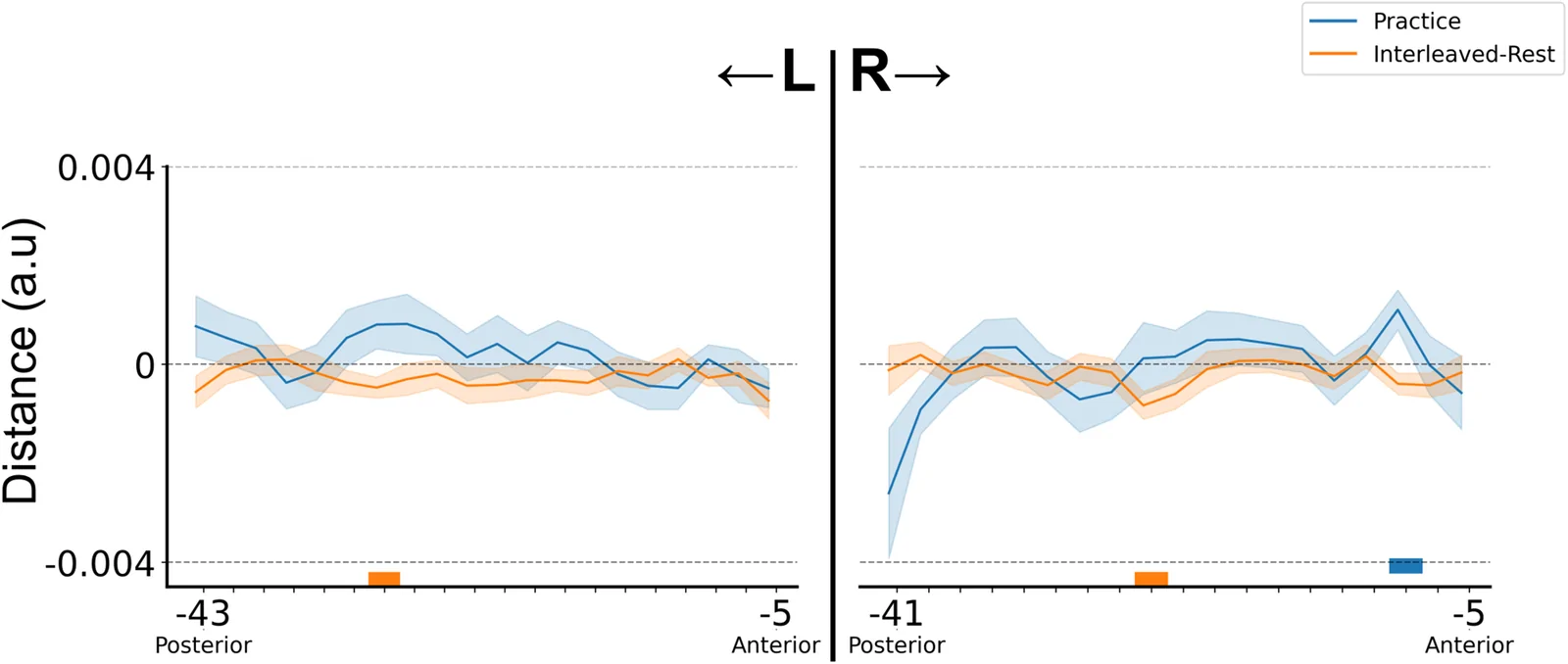
Profile ROI analysis of mean dissimilarity along the hippocampus The predefined hippocampal ROI from the Fan atlas was partitioned into slices along the *y* axis. Each point represents a participant’s mean dissimilarity value within a given slice, and the solid lines indicate the mean across participants, with shaded regions denoting ±SEM. Rectangles above the *x* axis mark slices exhibiting significant differences between practice and interleaved rest periods (uncorrected *p* < 0.05, two-sided *t* test).

### Persistence of neural patterns into post-learning rest

To probe the persistence of learning-related consolidation signals during interleaved rest, we used the MVCS method, which a recent fMRI study has successfully employed to demonstrate hippocampal reactivation.^13^ First, to isolate putative learning-related consolidation signals within the interleaved rest periods, we applied a generalized linear model (GLM) to remove task-evoked activity (background connectivity^23^). Next, MVCS^13^^,^^24^^,^^25^ analysis revealed that spatial activity patterns during interleaved rest more closely resembled the post-RS than the pre-learning resting state (pre-RS) in the ipsilateral M1/S1 and premotor cortex (Figures 4A and 4B, left) as well as the contralateral parietal lobule and M1 (Figures 4A and 4B, right). This persistence was also significant in the right putamen, contralateral to the practiced hand (*T*(24) = 3.55, uncorrected *p* = 0.002, false discovery rate [FDR]-corrected *p* = 0.006). No significant effect was observed in the left putamen (*T*(24) = 1.28, uncorrected *p* = 0.21; Figure 5A).

**Figure 4:**
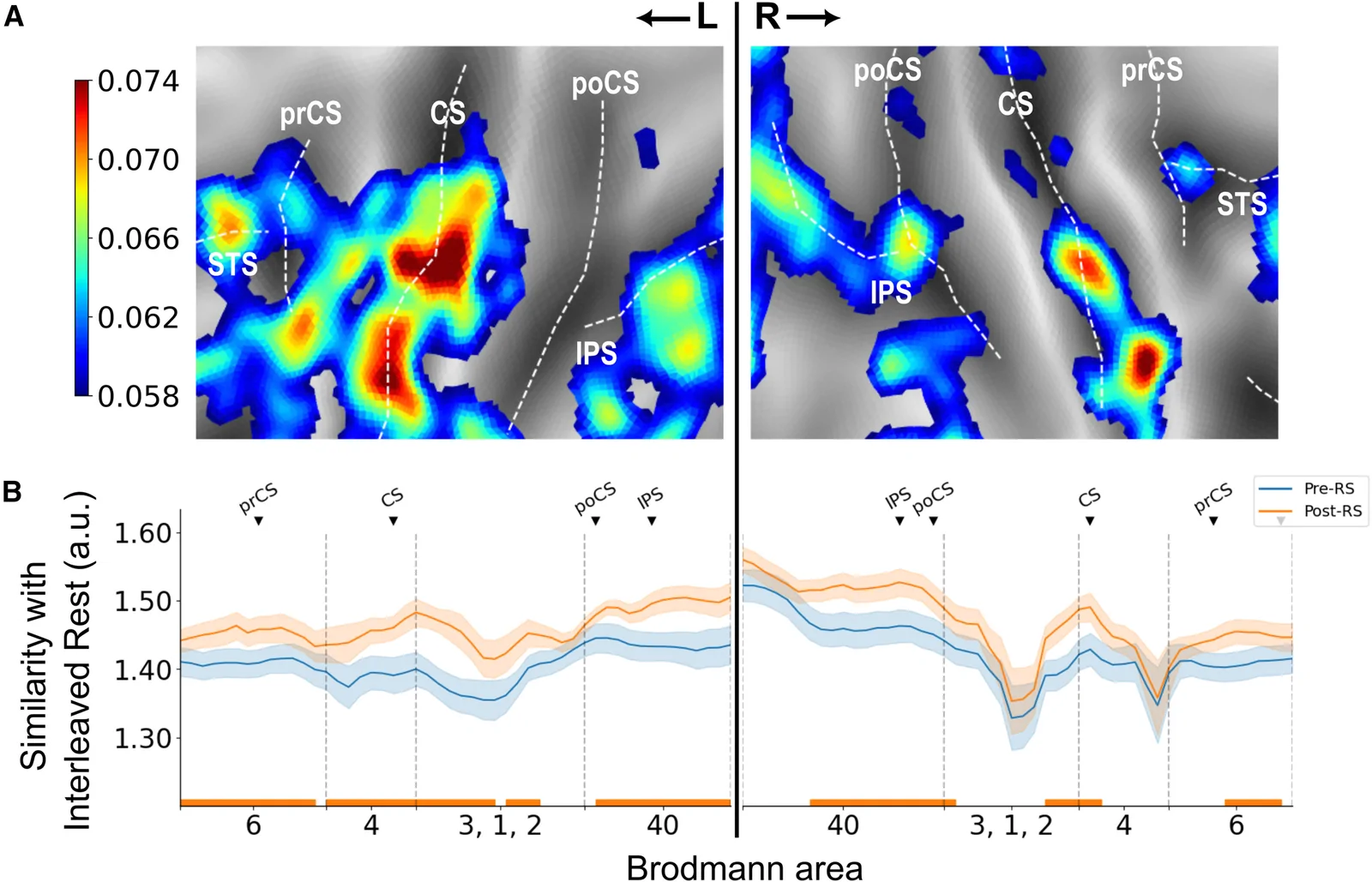
MVCS similarity analysis of patterns in sensorimotor areas (A) As shown in Figure 2, surface visualization of the brain illustrates the difference in similarity between the post-RS and interleaved rest, compared with the pre-RS and interleaved rest. The similarity values for post-RS and pre-RS were obtained using a whole-brain searchlight approach. Positive values indicate an increase in similarity value of the post-RS with interleaved rest compared with the pre-RS. Anatomical landmarks are labeled as follows: CS (central sulcus), prCS (precentral sulcus), poCS (postcentral sulcus), STS (superior temporal sulcus), and IPS (intraparietal sulcus). (B) Profile ROI analysis along the strip indicated in light purple above Figure 2A, showing the similarity values (±SEM) of the post-RS (orange) and pre-RS (blue) with the interleaved rest. The rectangles above the *x* axis indicate significant regions (uncorrected *p* < 0.05, paired two-sided *t* test).

**Figure 5:**
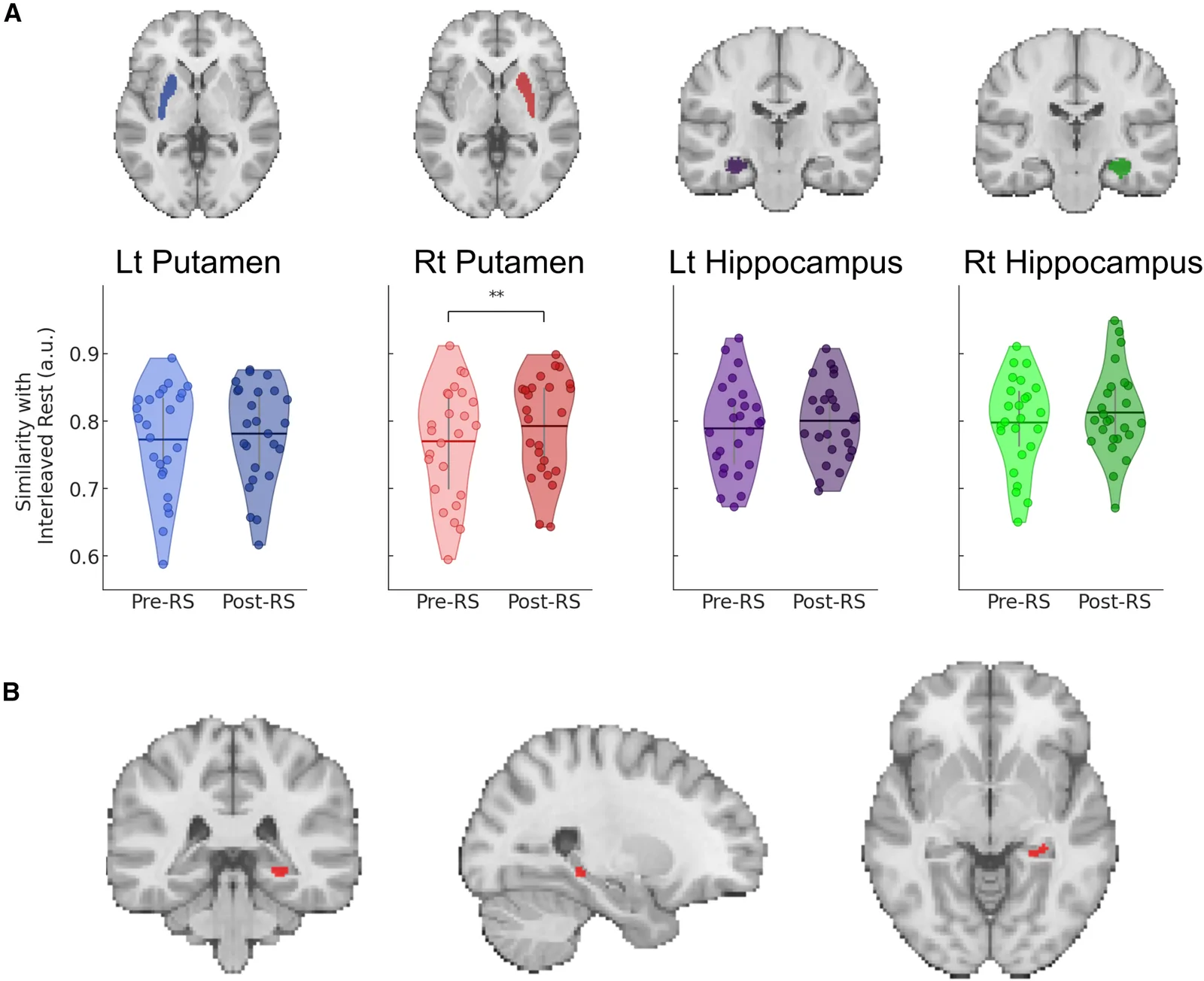
Similarity between interleaved rest during MSL and resting state in the hippocampus and putamen (A) Similarity between interleaved rest during the MSL task and the post-learning (post-RS) and pre-learning (pre-RS) resting states across the hippocampal and putamen ROIs. Similarity values were computed as Fisher z-transformed Pearson correlation coefficients. Dots within the violin plots represent individual participants’ similarity values. The first (25th percentile) and third (75th percentile) quantiles are indicated by the edges of the gray line, and the mean is represented by a solid line. ROIs from one representative subject are illustrated above each violin plot to show the regions used in the analysis. Statistical significance is indicated by ∗∗ *p* < 0.01, paired two-sided *t* test, FDR-corrected for multiple comparisons. (B) The searchlight analysis revealed a cluster of 40 voxels in the right posterior hippocampus, with small-volume correction (SVC) applied to the bilateral hippocampus. For statistical significance, we applied a voxel-wise threshold of *p* < 0.001 (uncorrected) and identified significant clusters using a cluster-wise *p* value threshold of *p* < 0.01. The searchlight analysis was performed using a spherical radius of 6 mm to assess the similarity between interleaved rest during the MSL task and the pre- and post-learning resting states at a finer spatial scale.

Similarly, MVCS patterns corresponding to active task practice (after explicitly modeling out the interleaved rest periods; see STAR Methods), more closely resembled post-RS than pre-RS activity. Consistent with interleaved rest findings, the task-MVCS patterns persisted during post-RS, although this effect exhibited distinct regional specificity at the ROI level. This persistence was particularly evident in the right putamen, contralateral to the practiced hand (*T*(24) = 3.60, uncorrected *p* = 0.001, FDR-corrected *p* = 0.005; Figure S6) as well as marginally significant for the right hippocampus (*T*(24) = 2.11, uncorrected *p* = 0.045, FDR-corrected *p* = 0.09; Figure S6). In contrast, there was no difference in similarity of the task-MVCS patterns with post-RS and pre-RS in the left putamen (*T*(24) = 1.25, uncorrected *p* = 0.22; Figure S6) and left hippocampus (*T*(24) = 1.38, uncorrected *p* = 0.18; Figure S6).

Next, to ensure that the enhanced correlations were not driven by temporal proximity, we investigated the dynamics of BOLD activity changes within interleaved rest. A mixed-effects linear model analysis of BOLD activity within interleaved rest (Table S1) revealed a significant main effect of Run (β = 0.006, *z* = 3.229, *p* = 0.001) and a significant interaction between Run and Condition (β = −0.012, *z* = −4.780, *p* < 0.001) in the right putamen. Simple slope analysis showed this interaction was driven by an increasing linear trend for the post-RS condition versus a decreasing linear trend for the pre-RS condition (Figure S5). A similar significant main effect of Run was found in the right hippocampus (β = 0.006, *z* = 2.951, *p* = 0.003), left hippocampus (β = 0.006, *z* = 3.090, *p* = 0.002), and left putamen (β = 0.004, *z* = 2.769, *p* = 0.006). Significant interactions between Run and Condition were also observed in these regions (right hippocampus: [β = −0.013, *z* = −4.609, *p* < 0.001]; left hippocampus: [β = −0.013, *z* = −5.009, *p* < 0.001]; left putamen: [β = −0.011, *z* = −4.876, *p* < 0.001]), indicating that similarity with post-RS gradually increased while similarity with pre-RS decreased.

To ensure that the observed increases in similarity between interleaved rest and post-RS were spatially specific to the task rather than driven by global signal drift or temporal proximity, we conducted a whole-brain spatial specificity analysis using the Brainnetome Atlas (Fan atlas).^26^ This analysis demonstrated that the vast majority of brain regions did not show a significant difference in similarity between the interleaved rest periods and pre/post-RS (Figure S7). The observed persistence of patterns is confined to specific learning-related networks (e.g., sensorimotor networks) and absent in valid control regions despite identical temporal conditions.

Although we found no evidence of rapid, sequence-specific hippocampal patterns during interleaved rest (Figure 3), an examination of the hippocampus showed a marginally greater similarity between interleaved rest and post-RS in the right hemisphere (*T*(24) = 1.93, uncorrected *p* = 0.065), with no significant effect in the left hemisphere (*T*(24) = 1.26, uncorrected *p* = 0.22; Figure 5A). A more sensitive searchlight analysis identified a localized cluster of 40 suprathreshold voxels in the right hippocampus (voxel-wise *p* < 0.001, cluster-wise *p* < 0.01, small-volume corrected; Figure 5B). This cluster showed significantly greater similarity between post-RS and interleaved rest than between pre-RS and interleaved rest.

## Discussion

Emerging evidence from recent studies suggests that the hippocampus, long thought to be independent of motor memories, contributes to MSL through the consolidation of motor memories during wakeful rest.^14^^,^^16^ In the current study, we hypothesized that the consolidation of motor sequences, a potential mechanism underlying consolidation, is supported by the reactivation of task-specific neural representations during wakeful rest. Based on this idea, we designed an experiment of learning multiple motor sequences simultaneously, distinguishing it from previous studies investigating the role of the hippocampus in motor learning. Using representational pattern analysis, we predicted that the unique multi-voxel activity patterns associated with different motor sequences would be distinguishable within the hippocampal BOLD activity during the subsequent interleaved rest.

A recent human study reported significant *MOGs* in the absence of *MOnGs*.^16^ Unlike prior work that focused on MOGs within a single repeated sequence, we employed a multi-sequence pseudorandom design^27^ to ensure that offline behavioral changes were not confounded by preparatory planning for an anticipated trial, even though this design induces strong anterograde and retrograde interference, which is known to attenuate early *MOGs* in MSL.^28^ Because adjacent trials in our design involved different, pseudorandomized sequences, the traditional within-sequence *MOGs* could not be computed. Therefore, to explicitly distinguish our measurement from standard metrics, we defined the adapted *MOG*_*as*_ to capture behavioral dynamics across the interleaved rest periods, regardless of sequence identity. We also introduced *TL*_*as*_ to evaluate the broader acquisition of motor skills.

In contrast to prior studies, we observed robust *MOnGs* accompanied by a negative *MOG*_*as*_. This negative *MOG*_*as*_ likely reflects the switch cost and behavioral interference inherent in alternating between different motor sequences in a pseudorandom environment. Due to the instability introduced by these factors affecting *MOG*_*as*_, it is difficult to definitely ascertain whether pure memory consolidation occurs during the immediate interleaved rest periods. Nevertheless, the fact that *TL*_*as*_ remained significantly positive despite this high interference environment is an intriguing phenomenon. It suggests that a sequence-independent representation may still emerge during the learning process. However, because these overall improvements span both practice and interleaved rest periods, it is difficult to attribute the acquisition of this sequence-independent representation exclusively to either phase. Future studies, designed to carefully disentangle online and offline periods, are necessary to clarify their specific contributions to sequence-independent learning.

Mean dissimilarity during the practice period was robust and statistically significant in the premotor and parietal regions, while it appeared more sparsely across the M1/S1 regions. These results align with a previous study that revealed a hierarchical representation of sensorimotor regions, with premotor and parietal regions processing higher-level information about motor sequences and chunks, and M1/S1 processing lower-level information about individual finger movements.^29^ Thus, the observed weak dissimilarity in the M1/S1 may be attributed to the effect of the first finger press, which differed across four sequences in our experiment, and not because of the whole sequence itself.^19^^,^^30^ M1/S1 also do not exhibit learning-related changes in pattern separation.^19^ Instead, the representation is shaped by the long-term natural hand use.^31^

Based on the view that hippocampal reactivation supports procedural memory consolidation^14^^,^^16^ and prior evidence that neural representations of sequences become more distinct after learning,^19^^,^^32^ we hypothesized that the brain encodes specific sequences by distinguishing them during practice. Our results show a dissociation between the neural representations of motor sequences during online execution versus offline rest, providing partial support for our initial hypothesis. Consistent with our predictions, sensorimotor regions exhibited robust sequence-specific activity patterns during the active practice period, confirming that distinct motor memories were successfully encoded. However, contrary to our prediction that these specific patterns would be maintained during offline periods, we did not observe direct evidence of sequence-specific discriminability in either the sensorimotor regions or the hippocampus during interleaved rest periods. This absence of sequence specificity during interleaved rest might indicate that offline consolidation does not rely on the simple replay of a single sequence. We propose two explanations to account for this lack of sequence-specificity. First, the pseudo-random presentation of multiple sequences creates high contextual interference, making it likely that memories of multiple sequences are reactivated during the same rest period,^11^^,^^33^ thus blurring sequence-specific patterns. Considering our highly interfering design, it remains possible that the hippocampus does not reactivate exact motor sequences but rather utilizes abstract reorganization to minimize interference among the different motor sequences. Through this process of abstract reorganization, cortico-hippocampal interaction reduces interference among overlapping sequences or even transfers knowledge from one sequence to another,^25^^,^^34^^,^^35^ thereby facilitating learning. This interpretation also aligns with Lin et al.,^36^ who reported a stronger functional connectivity between the premotor cortex and hippocampus under high contextual interference practice conditions, similar to those in our experiment. However, the absence of a fixed or repeated-sequence control condition in our study prevents a direct assessment of the underlying biological mechanisms. Second, while the pseudo-random trial order prevented deterministic planning of the upcoming sequence, it does not preclude probabilistic planning. Participants may engage in anticipatory planning for the next potential sequence. For example, after practicing sequence A, they might think that it’s more likely to have one of the remaining sequences (B, C or D), instead of sequence A again, which, given our pseudo-random order, would be a reasonable assumption for participants to make. This “probabilistic” planning activity could overlap with the memory reactivation signals of the previously learned sequence, reducing its distinctiveness during the interleaved rests.

Prior work has shown that neural representations formed during motor sequence practice persist into offline rest periods. King et al.^13^ found that multivoxel patterns in the hippocampus and putamen during post-RS reflected those observed during motor sequence practice, paralleling episodic-memory findings that hippocampal encoding patterns reappear during rest to support consolidation.^37^ While our study also investigates offline periods during interleaved rest, we observed a different outcome. To account for the lack of sequence-specific reactivation, we conducted an exploratory analysis comparing resting states to interleaved rest, which revealed the persistence of a generalized representational structure. Specifically, the representational geometry in the right putamen (contralateral to the practiced hand), bilateral sensorimotor and parietal cortices, and right posterior hippocampus more closely resembled post-RS than pre-RS. Considering these findings, we propose a post hoc interpretation and hypothesis: under an environment of high contextual interference, such as learning multiple motor sequences simultaneously, the hippocampal and sensorimotor systems may shift toward maintaining a generalized representation rather than distinct individual sequences during offline rest. While the hippocampus typically engages in pattern separation to keep similar sequences distinct,^35^ learning multiple sequences concurrently may necessitate a different mechanism to minimize interference and stabilize newly acquired skills. Currently, our data primarily supports the presence of this generalized state rather than direct evidence for the consolidation of an abstract representation. Altogether, these findings suggest that under this specific multi-sequence design, the hippocampal and sensorimotor systems maintain a generalized neural pattern during offline rest periods. However, it remains an intriguing possibility that concurrent learning of multiple sequences might also actively encourage the hippocampus to extract shared features across those sequences.^34^ Because our current design cannot definitively separate task-induced interference from a functional shift toward encoding abstract relational structures, this open question invites future research to explore a novel hypothesis concerning the role of the hippocampus and hippocampal replay in multi-task motor learning.

### Limitations of the study

Our current study has several limitations. First, we used fMRI to test the hypothesis about MSL and hippocampal replay. Hippocampal replay is known to occur during SWRs,^38^^,^^39^ which unfold on a compressed time scale relative to behavior.^40^^,^^41^ However, because the BOLD signal in fMRI has poor temporal resolution, it is not suitable for directly detecting SWRs, which are in the replay time window. Therefore, we adopted a naive approach by treating the whole interleaved rest period as the period in which hippocampal replay could occur. This naive selection of the replay time window may weaken the results of the mean dissimilarity analysis. To address this limitation, future studies should be conducted to simultaneously record electrical signals from electrodes implanted in the hippocampus and the BOLD signal.^42^ Second, we did not include a brain-behavior association analysis by collecting performance data through a post-learning retention test. Linking behavioral performance to the brain activity is essential for establishing the functional significance of the observed persistence of neural patterns. It could have given us valuable insight into the consolidation of motor memories. Lastly, the current experimental design, which lacks a control group that learns sequences in a fixed (repeated) order, does not allow us to assess interference or generalization among sequences directly. In addition, manipulating the level of contextual interference would be necessary to determine how hippocampal activity differentially contributes to memory consolidation under high versus low interference conditions.

## Resource availability

### Lead contact

Requests for further information and resources should be directed to and will be fulfilled by the lead contact, Sungshin Kim (sungshinkim@hanyang.ac.kr).

### Materials availability

This study did not generate new unique reagents.

### Data and code availability

• The unprocessed fMRI, refaced T1w, and behavioral data supporting the findings of this study have been deposited at a Zenodo repository (https://doi.org/10.5281/zenodo.22023537). In accordance with ethics approval and participant consent, which did not authorize public distribution, the data are placed under restricted access. The shared dataset is limited to 23 of the 25 original participants, as 2 individuals declined consent for their data to be shared externally. Data access is restricted to qualified researchers for academic purposes and can be requested via Zenodo or by contacting the lead contact.
• The custom Python package boostrsa (built upon rsatoolbox) is available on GitHub: https://github.com/SeojinYoon/boostrsa and deposited at Zenodo under https://doi.org/10.5281/zenodo.21806632. The analysis and visualization codes are available on GitHub: https://github.com/SeojinYoon/Motorconsolidation and deposited at Zenodo under https://doi.org/10.5281/zenodo.22060276.
• Any additional information required to reanalyze the data reported in this paper is available from the lead contact upon reasonable request.

## Acknowledgments

This work was supported by the Institute of Information and Communications Technology Planning and Evaluation (IITP) grant funded by the Korea Government (MSIT, Ministry of Science and ICT) (grant no. RS-2026-25548925) and the Korea Health Technology R&D Project through the Korea Health Industry Development Institute (KHIDI), funded by the Ministry of Health and Welfare, Republic of Korea (HI19C075300).

## Author contributions

S.K. conceived the idea presented in the study. S.Y. and S.K. designed the experiment. S.Y. carried out the experiment. S.Y. and A.C. performed formal analysis; S.Y., A.C., and S.K. wrote the manuscript.

## Declaration of interests

The authors declare no competing interests.

## Declaration of generative AI and AI-assisted technologies in the manuscript preparation process

During the preparation of this work, the authors used ChatGPT4.0 and Grammarly in order to edit the manuscript with grammar checks. After using these tools, the authors reviewed and edited the content as needed and take full responsibility for the content of the publication.

## STAR★Methods

### Key resources table

**Table:** 

REAGENT or RESOURCE	SOURCE	IDENTIFIER
**Deposited data**		

Raw and analyzed data	This paper	https://doi.org/10.5281/zenodo.22023537

**Software and algorithms**		

AFNI (Analysis of Functional NeuroImages)	https://afni.nimh.nih.gov/	RRID: SCR_0052927
FSL	https://www.fmrib.ox.ac.uk/fsl/	RRID: SCR_022823
FreeSurfer	https://surfer.nmr.mgh.harvard.edu/	RRID:SCR_001847
Psychopy	https://github.com/psychopy/psychopy	RRID: SCR_006571
Boostrsa	https://github.com/SeojinYoon/boostrsa	https://doi.org/10.5281/zenodo.21806632
surfAnalysisPy	https://github.com/DiedrichsenLab/surfAnalysisPy	N/A
MotorConsolidation	https://github.com/SeojinYoon/MotorConsolidation	https://doi.org/10.5281/zenodo.22060276

**Other**		

MRI 3T	Siemens, Prisma with a 64-channel head coil	N/A
8 Buttons Bimanual	Cambridge Research Systems	N/A

### Experimental model and study participant details

#### Participants

Twenty-five neurologically healthy and right-handed subjects (8 females; ages: mean ± SD = 25.3 ± 3.2, 19–35 years) participated in the study. They provided written informed consent and received compensation for their participation. The study protocol was approved by the Institutional Review Board of Sungkyunkwan University, Suwon, Republic of Korea (IRB No. 2018–05-003–032), and all research methods were performed in accordance with the Declaration of Helsinki.

### Method details

#### Experiment design

Participants were instructed to press four buttons with the fingers of their non-dominant left hand, following the visually presented sequence as quickly and accurately as possible. The right dominant hand is prone to a faster ceiling effect than the non-dominant left hand, by using the non-dominant hand, we can focus on a longer learning activity where we can observe the fast consolidation processes prior to the performance plateau. In the event of an error during sequence execution, they were instructed to resume from the element at which the mistake occurred or restart the sequence from the beginning. When participants completed a sequence, an asterisk appeared below the sequence of numbers to motivate them to improve performance. We also instructed them to earn as many asterisks as possible. To ensure participants understood the instructions, they performed a behavioral test 1-2-3-4-1 outside the MRI scanner before the experiment. We confirmed that they pressed the buttons following the sequence multiple times and stopped pressing them during the rest periods. The entire fMRI experiment consisted of two resting state runs, one before and one after four motor sequence learning task runs (Figure 1A). The motor sequence learning task consisted of four runs, each comprising 24 trials (96 trials in total), and lasted approximately 50 min. For each trial, one of four 8-element sequences (1-4-2-3-1-2-4-3, 2-1-3-4-2-3-1-4, 3-4-2-1-3-2-4-1, 4-1-3-2-4-3-1-2, 1 being the pinky finger and 4 being the index finger) was presented for 10 s and followed by a rest period of 20 s with a fixation cross on the center of the screen. We intentionally chose a long inter-stimulus interval (ISI) to ensure enough time for pattern reactivation during the rest periods, allowing us to investigate whether pattern reactivation occurs within that time frame. Additionally, this choice allows for a more reliable estimation of the activity pattern during the rest period while being less affected by the delayed hemodynamic response from the preceding practice period. Significantly, the slow block design would reduce artifacts in neural representational similarity analysis.^43^^,^^44^ Four different sequences were presented in a pseudorandom order in a task block, so all four sequences were presented in four consecutive trials (Figure 1A).

#### MRI acquisition

We collected fMRI image data using a 3T MRI scanner in the Center for Neuroscience Imaging Research (CNIR) at Sungkyunkwan University, equipped with a 64-channel head coil. An ear plug and a foam pad were used to reduce noise from fMRI and participants’ head motion. Functional scans during four runs were acquired by using an echo planar imaging (EPI) sequence with the following spec: repetition time (TR) = 2000 ms; voxel size = 2.0 × 2.0 × 2.0 mm; echo time (TE) = 30 ms; field of view (FOV) = 200 mm; flip angle (FA) = 90°; slice thickness = 2.0 mm; matrix = 96 × 114 × 96 voxels; the number of time steps = 363. Following each of the functional runs, two EPI images with opposite-phase directions (posterior-to-anterior) were acquired for distortion correction. For anatomical reference, a whole-brain T1-weighted anatomical scan was performed using a magnetization-prepared rapid acquisition with gradient echo MPRAGE sequence, with the following parameters: TR = 2300 ms; voxel size = 1.0 × 1.0 × 1.0 mm; TE = 2.28 ms; FOV = 256 mm; FA = 8°; slice thickness = 1.00 mm; matrix = 193 × 229 × 193 voxels.

#### fMRI data preprocessing

The T1 and fMRI data were preprocessed following a standard procedure suggested by Analysis of Functional NeuroImages (AFNI) software.^45^ The T1 images and functional echo planar image (EPI) data were transformed into volume data using AFNI’s “Dimon” function and realigned from oblique to cardinal orientation using AFNI’s “3dWarp”. The inhomogeneity in the T1 image due to magnetic field bias was corrected, and the skull was removed using AFNI’s “3dUnifize” and “3dSkullStrip”, respectively. Each participant’s brain was transformed into a standard Montreal Neurological Institute (MNI) template using AFNI’s “@auto_tlrc” function. For functional data, spikes were removed using AFNI’s “3dDespike” to reduce outlier effects. Then, time correction was applied to minimize time differences between each slice using AFNI’s “3dTshift”. An affine transformation was used to adjust motion-related artifacts using AFNI’s “3dAllineate”. All EPI data had a resolution of 2.0 × 2.0 × 2.0 mm. For both resting-state functional scans (pre-learning and post-learning), we performed additional preprocessing. The realignment parameters computed during the preprocessing step were used to compute the Framewise Displacement (FD^46^). If any volume *n* had an FD exceeding 0.4 mm, the volumes *n* and *n−*1 were removed from the analyses. The percentage of volume excluded from the pre-learning and post-learning resting state scans was 0.74 (SD = 1.65) and 1.03 (SD = 1.89), respectively.

To delineate cerebrospinal fluid (CSF), white matter (WM), and gray matter (GM), we employed the high-resolution T1-weighted scan of each subject after preprocessing. The segmentation tool from FMRIB’s Automated Segmentation Tool (FSL FAST^47^) was used to generate partial volume maps. The CSF and WM masks were set to include only voxels with a probability of 1 from the partial volume maps. This ensures that only voxels belonging to these tissues were used to compute the principal components using AFNI’s ‘3dpc’. The principal components were computed on preprocessed, censored, detrended, and high-pass filtered data to prevent any influence of these steps on the principal components. Moreover, the CSF and WM fMRI data were z-scored prior to computing the principal components. Nuisance regressors encompass the first three principal components of the CSF and WM, six rigid-body transformations, along with their first derivatives,^48^^,^^49^^,^^50^^,^^51^ yielding 12 motion regressors. Additionally, detrending and high-pass filtering with a cutoff frequency of 0.08 Hz were applied and integrated into the regression analysis. Filtering was performed using sine and cosine basis functions to minimize artifacts from censoring.

### Quantification and statistical analysis

#### Activation maps for pattern analysis

To calculate the BOLD activation level for each sequence, separate GLM analyses were performed for the practice and rest periods. Having two regressors encoding both periods in a single GLM could cause a multicollinearity problem due to the overlap of regressors convolved with the hemodynamic response. As such, GLM analyses were conducted separately on the practice and rest periods to address this issue. The GLM for the practice periods included four regressors encoding different sequences separately and regressors of non-interest, including six regressors related to head motions and third-order polynomials modeling fMRI signal drifts. The GLM for the rest periods was designed similarly, except for four regressors encoding different rest periods following the motor sequences practice. GLM analyses were performed separately for each of the four fMRI runs, resulting in eight activation maps: four for the practice periods and four for the rest periods.

#### ROI selection

To perform profile ROI analysis from the premotor cortex to the parietal lobule, we defined a diagonally oriented virtual strip to minimize overlap between the SPL and IPL. This strip was subdivided into 30 rectangular sections of equal length along the virtual strip and a fixed surface height of 20 units on the FreeSurfer^52^ template surface, which contains approximately 32,000 vertices per hemisphere. The number of vertices per rectangle varied depending on its location on the surface, with an average of 68 vertices (SD = 9.13; range = 51–86; Figure S3).

For the performance profile analysis, we partitioned the predefined hippocampal ROI from the Fan atlas into 20 slices along the y axis for each hemisphere (Figure S2). For each slice, we assessed voxel coverage relative to each participant’s full-brain mask and retained only those slices that contained at least 10 voxels within the mask. This procedure yielded 20 slices in the left hemisphere and 19 slices in the right hemisphere of the hippocampus.

To define subject-specific ROIs for the hippocampus and putamen, we used FMRIB’s Integrated Registration Segmentation Toolkit (FSL FIRST^53^) on each subject’s preprocessed T1-weighted scan. This approach yielded volumetric segmentations of four ROIs: the left and right hippocampus, as well as the left and right putamen. To further refine these ROIs, we retained only voxels with a probability greater than 10% of belonging to the GM. The final ROIs contained an average of 596 voxels (SD = 82; range: 394–744) in the left hippocampus, 620 voxels (SD = 81; range: 465–753) in the right hippocampus, 799 voxels (SD = 81; range: 688–1019) in the left putamen, and 800 voxels (SD = 81; range: 634–981) in the right putamen.

#### Representational pattern analysis

To identify motor sequence representations across sensorimotor regions, we employed representational pattern analysis using a searchlight approach. We defined spherical ROIs (searchlights) spanning the whole brain^54^ with a radius of 6 mm (93 nearest voxels from the center, 744 mm^3^) within the brain mask. For each searchlight, we constructed a representational dissimilarity matrix (RDM) by computing the multivariate distance between pairs of activation maps related to four sequences during practice or resting periods, yielding an 8 × 8 RDM. For the distance metric, we selected a cross-validated Mahalanobis distance, which is unbiased and reliable due to cross-validation and multivariate noise normalization.^22^ The multivariate noise normalization was performed separately for each activation map derived from the practice and interleaved rest conditions by estimating the covariance from the GLM fitting residuals and regularizing it through Ledoit-Wolf optimal shrinkage.^55^

Using these RDMs, we derived two key measures. First, mean dissimilarity was computed by averaging the pairwise dissimilarities among all pairs of sequences within each period (practice or rest). Second, to quantify a reactivation index, we compared activity patterns between the practice and interleaved rest periods. Although same-sequence dissimilarity is expected to decrease if reactivation occurs, differences in overall brain state between practice and interleaved rest inflate these values. To account for this, we established a baseline defined as the mean dissimilarity of different-sequence pairs across practice and rest. The reactivation index was then defined as the negative difference between the same-sequence dissimilarity and this baseline, isolating sequence-specific reactivation from global state-dependent effects (Figure S2).

For the profile ROI analysis^56^ in the sensorimotor regions (Figure 2), we first transformed the volumetric mean dissimilarity maps into surface mean dissimilarity maps using the surfAnalysisPy toolbox (https://github.com/DiedrichsenLab/surfAnalysisPy). For each rectangle, we averaged the vertices and assessed the statistical significance of mean dissimilarity by conducting a two-sided *t* test across 25 participants to determine whether the mean dissimilarity is significantly larger than zero.^57^

#### Multi-voxel correlation structure (MVCS) analysis

To assess how MSL influences the correlation structure between voxels in the hippocampus and putamen over a longer timescale, we compared the multi-voxel patterns from the pre-learning resting-state (pre-RS) and post-learning resting-state (post-RS) with the task-based functional scans. To minimize processing-induced biases, we applied the same denoising strategies to the task-based scans as we did to the resting-state functional scans. These included high-pass filtering with a cutoff at 0.008 Hz, detrending, scrubbing (censoring excessive motion volumes), and nuisance regression (motion parameters, first derivatives, first three principal components of CSF and WM). For the task-based scans, the percentages of volume excluded from the first, second, third, and fourth runs were 2.86 (SD = 6.32), 3.83 (SD = 8.37), 4.88 (SD = 10.58), and 4.13 (SD = 6.71), respectively. To preserve spatial information and avoid contamination from neighboring regions when comparing voxel-by-voxel matrices between different conditions, we did not apply spatial smoothing.

To ensure that pattern estimates were not contaminated by the temporal adjacency between task practice and interleaved rest, we created two separate GLMs to estimate orthogonal neural patterns. For the task practice pattern, we explicitly modeled out the interleaved rest periods via four dedicated regressors (one for each followed sequence) to ensure the residuals reflected only task activity. For the interleaved rest pattern, we modeled out the four task sequences as nuisance regressors, ensuring that only the interleaved rest periods contributed to the residual pattern. The residual time series of those orthogonal GLMs were used to compute the MVCS matrices, using procedures identical to those in previous works.^13^^,^^37^

MVCS analysis was performed separately for each ROI and each functional scan of interest (pre-RS and post-RS and MSL task scans). For each resting-state scan, the MVCS was computed by calculating Pearson’s correlation between the time series of each voxel within an ROI, resulting in a voxel-by-voxel correlation matrix. Regarding the MSL task scans, a two-step approach was employed to aggregate data across the four runs. Firstly, the BOLD time series for each voxel was z-scored within each run and then concatenated across the four runs to compute a single MVCS matrix. To assess the pattern persistence, we computed the similarity (Pearson correlation) between the Fisher’s z-transformed upper triangles of the task-MVCS matrix and the resting-state MVCS matrices (pre-RS and post-RS). Following this procedure, for each subject and each ROI, the resulting similarity values were z-scored to facilitate statistical analyses. Paired two-sided t-tests were conducted to compare the similarity values of pre-RS and MSL task scans, as well as between post-RS and MSL task scans. To account for multiple ROIs, a false discovery rate (FDR) correction^58^ was applied across the four ROIs.

To identify the dynamics of changes in neural patterns, a linear mixed-effects model was used to predict the similarity value between interleaved rest and pre/post-RS in the following form:

Similarity∼Run∗Condition+(1|Participant)

where Run represents the run number (1–4), Condition corresponds to either the comparison between the interleaved rest and pre-learning rest (Pre) or between interleaved rest and post-learning rest (Post), and (1|Participant) reflects the random intercept assigned to each participant. In addition to the ROI-based analysis, we conducted an exploratory whole-brain searchlight analysis to identify localized patterns that might not be detectable at the broader ROI level. We implemented spherical searchlights with a 6 mm radius, including the 93 nearest voxels from the center (744 mm^2^). All voxels within each sphere were required to fall within the whole-brain mask. For each spherical ROI, we applied the same MVCS methodology as previously described to generate two z-scored similarity maps: one representing the similarity between pre-RS and MSL task, and the other between the post-RS and MSL task. We first examined the resulting maps in the sensorimotor regions, followed by the hippocampus and putamen. In the sensorimotor regions, we used the same rectangular ROI profile approach as described earlier to compare pre-RS and post-RS similarity values. Specifically, we averaged the similarity value within each of the rectangular ROIs. Then, we conducted a paired *t* test across the 25 participants to assess the persistence of interleaved rest patterns in the post-RS period compared to the pre-RS period (*p* < 0.05). Given our strong *a priori* hypothesis regarding the role of the hippocampus in motor memory consolidation, we applied small-volume correction within an anatomically defined bilateral hippocampus mask.^26^ For cluster-wise multiple comparison correction, we used Monte Carlo simulations (AFNI’s “3dttest++” with the “-Clustsim” option), with a voxel-wise threshold of *p* < 0.001. Based on the simulation, the minimum cluster size required for significance at *p* < 0.01 (cluster-corrected) was 18 voxels (144 mm^3^) connected through faces, edges, or corners.

To ensure that the observed increase in pattern similarity between the interleaved rest periods and post-RS was driven by learning-specific consolidation rather than non-specific temporal proximity (i.e., global signal drift causing temporally adjacent time-points to exhibit higher correlation), we performed a whole-brain control analysis. We employed the Brainnetome Atlas (Fan atlas) to segment the brain into 280 regions of interest (ROIs). After applying a subject-specific gray-matter mask, ROIs with insufficient voxel coverage (<10 voxels) were excluded. If even one subject had an insufficient number of voxels for a specific ROI, we removed this ROI for all the subjects. This resulted in 270 valid ROIs. For each region, we computed the MVCS similarity between the acquisition patterns and the pre- and post-RS. This allowed us to determine whether the pattern persistence (Post-RS > Pre-RS) was a spatially specific effect confined to the motor network or a global confound affecting unrelated control regions.

#### Performance measurement

To quantify motor skill acquisition, performance (correct sequence typing speed) was measured in sequences per second (seq/s). Data processing was restricted exclusively to fully correct sequence completions (8 successive correct presses), with all attempts containing errors being excluded from the analysis. For each successfully completed sequence, the 7 inter-tap intervals (ITIs) were computed. Performance for that sequence was defined as the inverse of its estimated total duration, which was calculated by multiplying the mean ITI by 7. Trial performance for each participant was calculated by averaging the seq/s values across all successful sequence completions within that trial (Figure S1). Block performance was then obtained by averaging the correct sequence speed across the four different sequences presented within a given block.

A two-way repeated measures analysis of variance (ANOVA) was performed to examine two objectives. First, we aimed to assess whether participants correctly learned to perform the four distinct sequences by analyzing the change in speed performance across blocks. Second, we investigated whether there were significant differences in speed performance among the four sequences. The factors included blocks (from the first to the last, totaling 24) and the four distinct sequences, with speed performance as the outcome measure. Both blocks and sequences were within-subject factors, and the interaction between them was tested. Greenhouse-Geisser corrections were applied to adjust for potential violations of the sphericity assumption in the repeated measures design.

The learning phase corresponds to the period in which performance improves rapidly. Here, it is defined as the set of trials from the first trial of the first block to the last trial of the cutoff block. Similarly to a previous study,^16^ we determined the cutoff block by fitting an exponential model of the following form to each subject’s block speed data (average of the correct sequence speed for all the trials in a block):

$$L\;\left(b\right)=C_{1}+C_{2}\;\cdot \;1-e^{\lambda b}$$

where b represents the block number, *C*_1_ represents pre-training performance, *C*_2_ controls the asymptote (*C*_1_ + *C*_2_) and *λ* controls the learning rate. The cutoff block was defined as the first block where 95% of the learning had been achieved, separately for each subject. On average, the cutoff block was the 15^th^ block (SD = 5.74, range: 4–22).

Individual performance improvement was assessed by computing micro-online gains (*MOnG*), micro-offline gains across sequences (*MOG*_*as*_), and total learning across sequences (*TL*_*as*_). *MOnG* was defined as the performance (seq/s) difference between the first and last correctly completed sequences of a trial. Regarding offline gains, *MOG*_*as*_ was defined as the performance difference between the last correct sequence completion of a practice period and the first correct sequence completion of the subsequent period. *TL*_*as*_ was defined as the performance difference between the first correct sequence completion of a practice period and the first correct sequence completion of the subsequent period. Both *MOG*_*as*_ and *TL*_*as*_ were computed irrespective of sequence condition. To ensure data quality, we applied rigorous filtering over each participant’s data where trials were included only if the respective sequences were correctly completed. This resulted in average exclusion rates of 10% for *MOnG* (SD = 7.00, range: 2.63–29.76), 10% for *MOG*_*as*_ (SD = 5.75, range: 2.67–25.37), and 14% for *TL*_*as*_ (SD = 7.86, range: 4.00–34.33). After calculating each trial’s *MOnG*, and *MOG*_*as*_, we averaged the results across trials up to the asymptote block to prevent the dilution of gain estimates by the performance plateau.

In addition, although it was in principle possible to compute micro-offline gains within the same sequence (*MOG*_*ws*_) when identical sequences occurred consecutively, as in prior work,^15^ the number of such instances was limited. Across participants, the mean number of consecutive same-sequence trials was 5.12 (SD = 1.86, range = 2–8), and only 9 of the 25 participants exhibited valid consecutive occurrences for all four sequences. Moreover, each sequence was repeated consecutively only a small number of times (range = 1–3), with mean consecutive occurrences of 1.77 (SD = 0.91) for Seq1, 1.66 (SD = 0.99) for Seq2, 1.77 (SD = 0.41) for Seq3, and 1.33 (SD = 0.47) for Seq4. Given this limited number of within-sequence repetitions, the dataset was insufficiently powered to reliably estimate *true* MOG; therefore, these analyses were not pursued.

Statistical analyses were conducted using Python (version 3.10.19), AFNI’s 3dttest++ (version 25.2.05). All statistical details of the analyses, including the specific tests used (e.g., two-way repeated measures ANOVA, two-sided t-tests, and linear mixed-effects models), can be found in the Results section, figures and figure legends. For all analyses, the exact value of *n* is 25, which represents the number of participants. Throughout the manuscript, the definition of center is the mean, and dispersion measures are represented as the standard error of the mean (SEM) for behavioral data and the standard deviation (SD) for demographics, voxel counts and volume counts.
