# Supplementary material for: Neural substrates related to memory consolidation of learning multiple motor sequences during wakeful rest
Source: iScience. 2026 Oct 26;29(11):117653. doi: 10.1016/j.isci.2026.117653 (PMC13734795; doi:10.1016/j.isci.2026.117653)
Supplement: Supplementary file 1 [file mmc1.pdf]

iScience, Volume *29*

Supplemental information

Neural substrates related to memory consolidation of learning multiple motor sequences during wakeful rest

Seojin Yoon, Antoine Caraballo, and Sungshin Kim

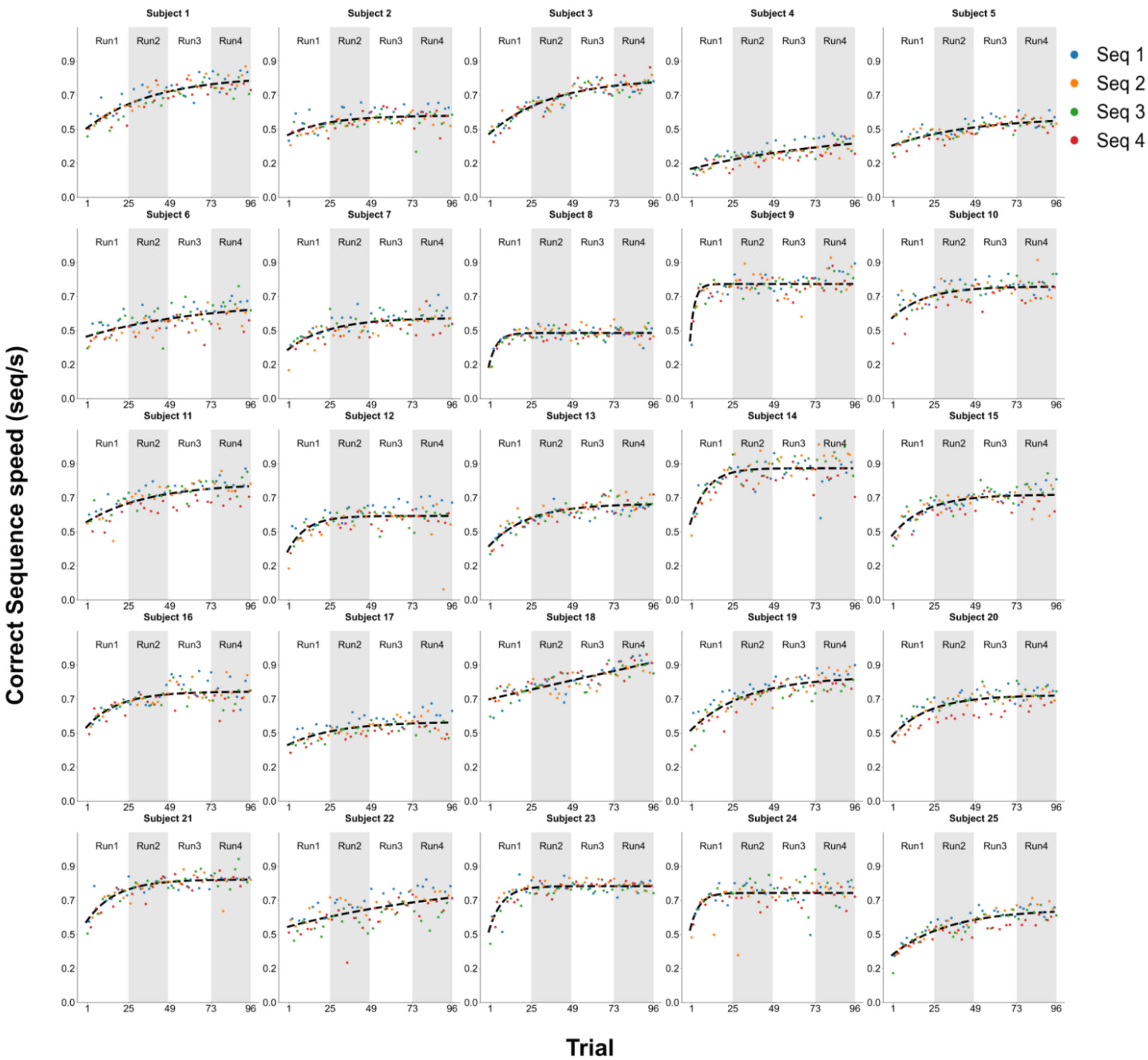

Figure S1. Learning curves of 25 individual participants, Related to Figure 1

For all 25 participants, performance, measured as the speed of correct sequence completion, improved across multiple task blocks for the four sequences.

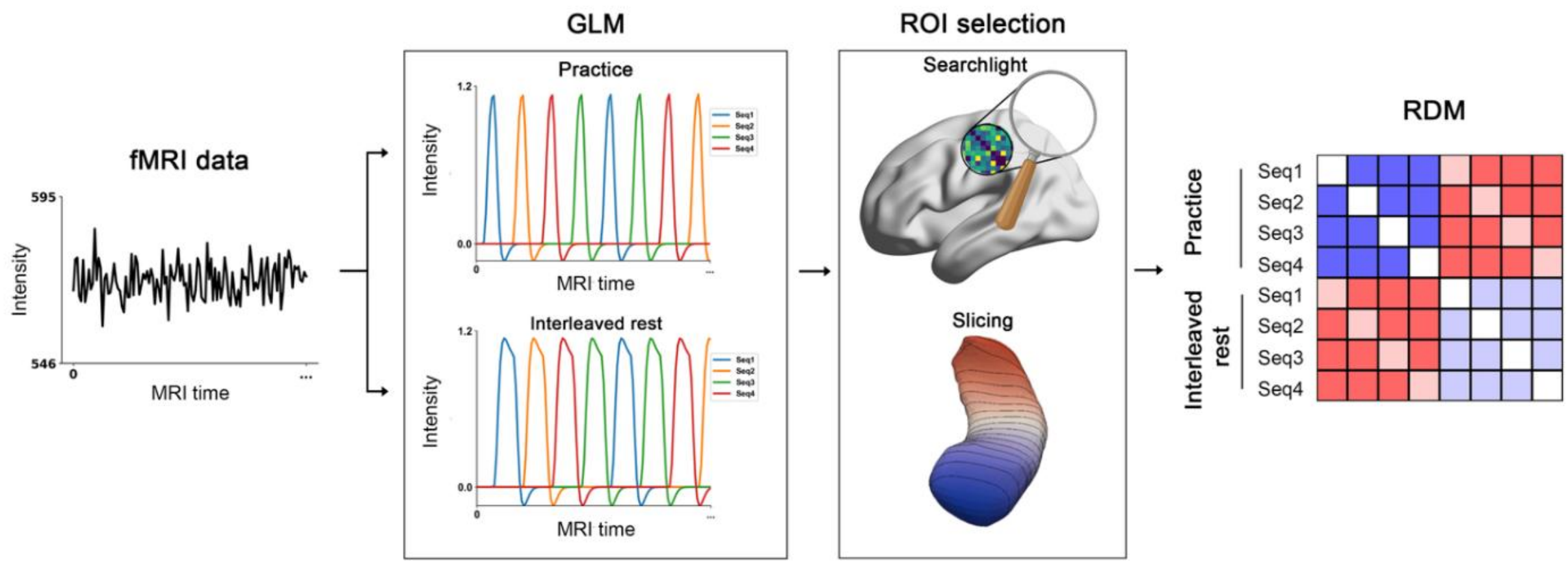

Figure S2. Representational pattern analysis, Related to Figure 2

A General Linear Model (GLM) was applied to the raw fMRI time series to estimate sequence-specific BOLD activation patterns for both practice and interleaved rest periods separately, convolved with a canonical hemodynamic response function. Based on these beta estimates, Representational Dissimilarity Matrices (RDMs) were computed using multivariate voxel patterns defined by two distinct ROI approaches: a searchlight analysis utilizing a moving spherical window to scan the entire brain, and a slicing method wherein the hippocampus was segmented along its longitudinal axis (posterior to anterior) to analyze patterns on a slice-by-slice basis. Finally, four types of mean dissimilarity measures were derived from the RDMs: within practice dissimilarity (blue), within interleaved rest dissimilarity (light blue), cross-state (practice and interleaved rest) same-sequence dissimilarity (light red), and cross-state different-sequence dissimilarity (red). Specifically, the reactivation index was defined as the negative difference between the mean cross-state same-sequence dissimilarity and the mean cross-state different-sequence dissimilarity.

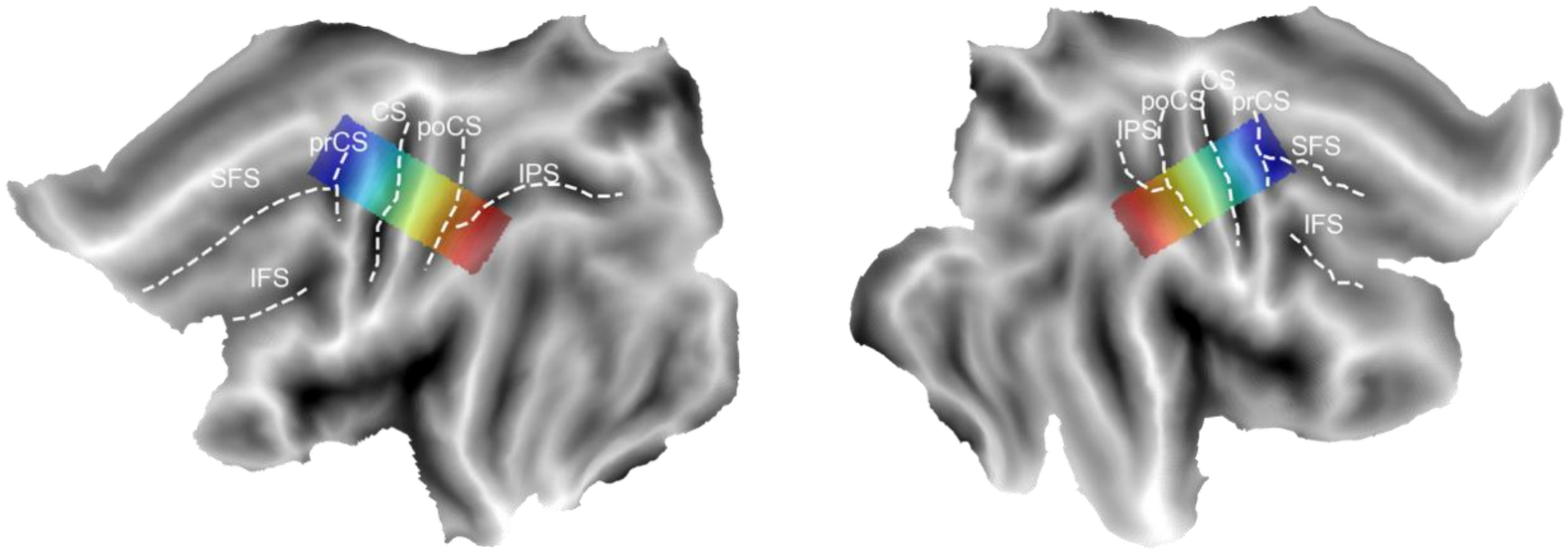

Figure S3. Profile ROI analysis, Related to Figure 2 and 4

Profile ROI analysis on the cortical surface. Regions of interest are defined along the specific trajectory, with color gradients representing variations of ROI values. The trajectory extends from the premotor area to the inferior parietal lobule and is divided into 30 rectangular ROIs of equal length. The mean dissimilarity within each rectangle was averaged and displayed in Figure 2B. The dotted lines indicate the names of sulci.

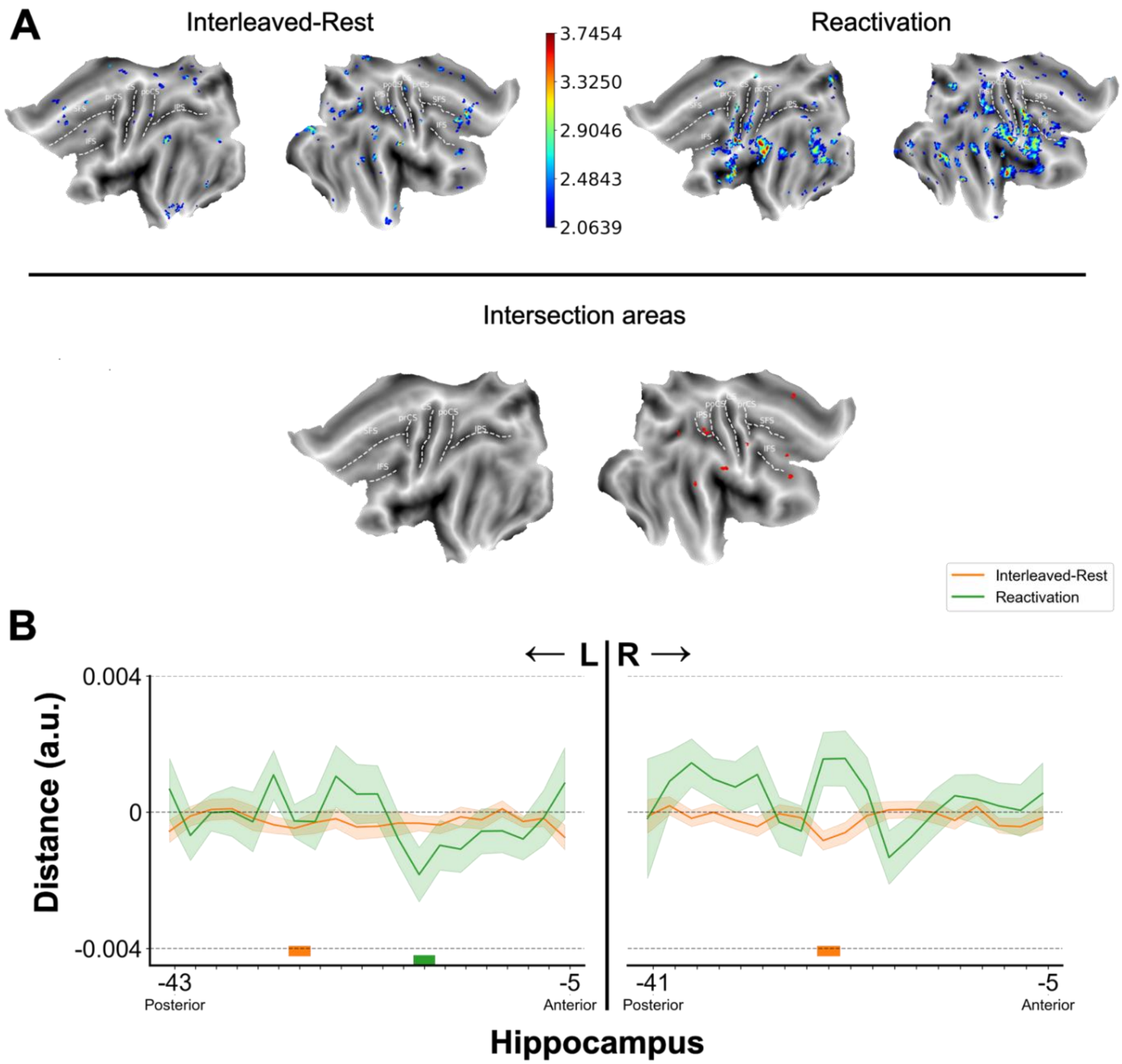

Figure S4. Analysis of sequence-specific reactivation between task and interleaved rest periods, Related to Figure 2

(A) Whole-brain analysis. The top row displays the mean dissimilarity during interleaved rest (left) and the reactivation index (right). The reactivation index represents the reduction in dissimilarity for same-sequence pairs relative to different-sequence pairs. Areas with significant positive values ($T(24) >= 2.0639$, $p <= 0.05$, two-sided t-test) are shown. The bottom row shows an intersection map highlighting the spatial overlap between significant interleaved rest period dissimilarity and the reactivation index. (B) Hippocampus ROI analysis. The same analysis was applied to the sliced hippocampus. Significant areas ($p < 0.05$, two-sided t-test) are indicated by rectangles. Data are represented as mean ± SEM.

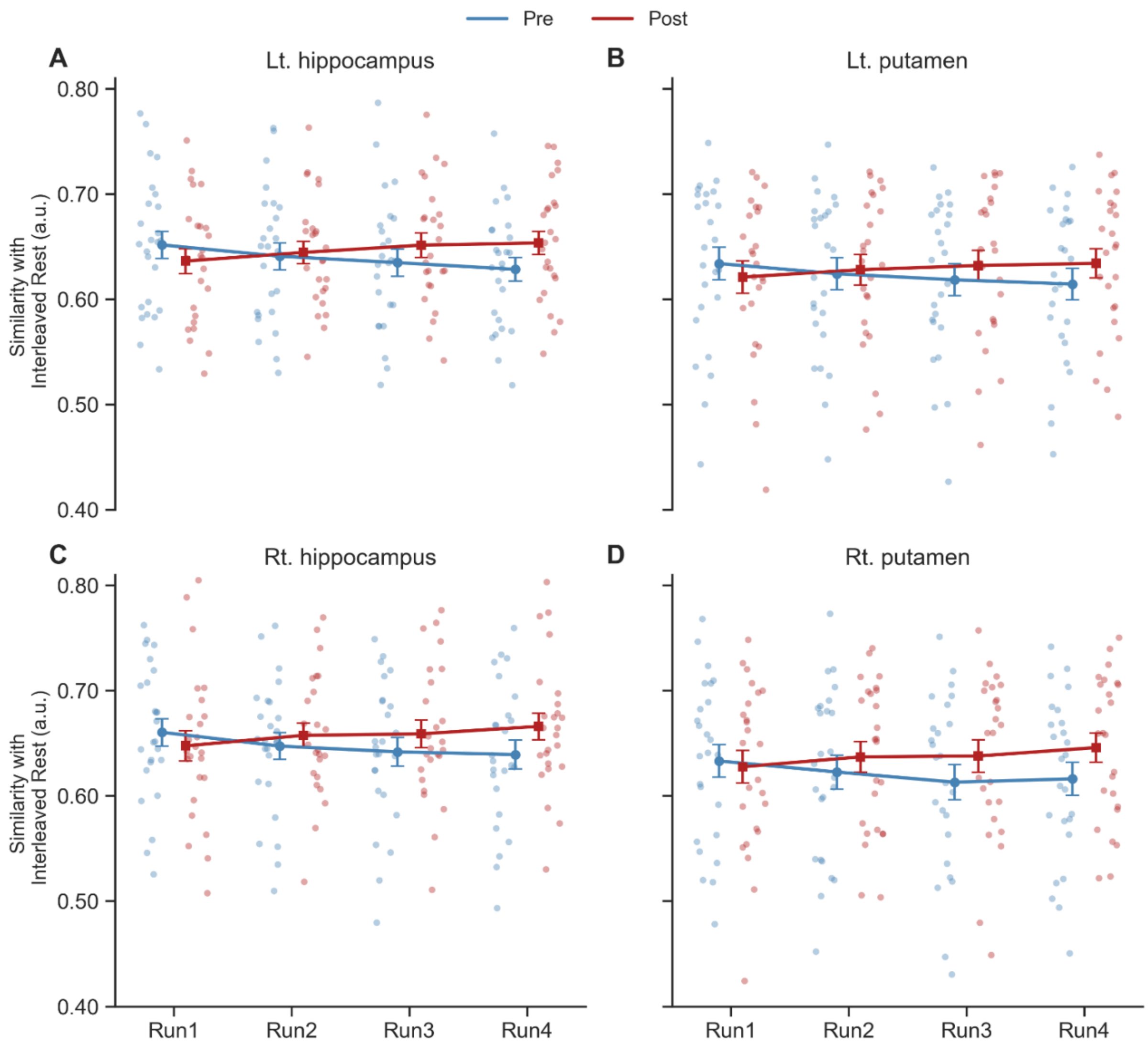

Figure S5. Dynamics of similarity values (SI) between interleaved rest and Pre/Post-learning rest across runs, Related to Figure 5

Changes in MVCS similarity values over the experiment for four ROIs. (A) Left Hippocampus, (B) Left Putamen, (C) Right Hippocampus, and (D) Right Putamen. Each panel displays the individual data points as colored dots and the mean similarity values (red squares and blue circles with error bars representing mean ± SEM) for both Pre-learning (blue) and Post-learning (red) rest comparison with interleaved rest.

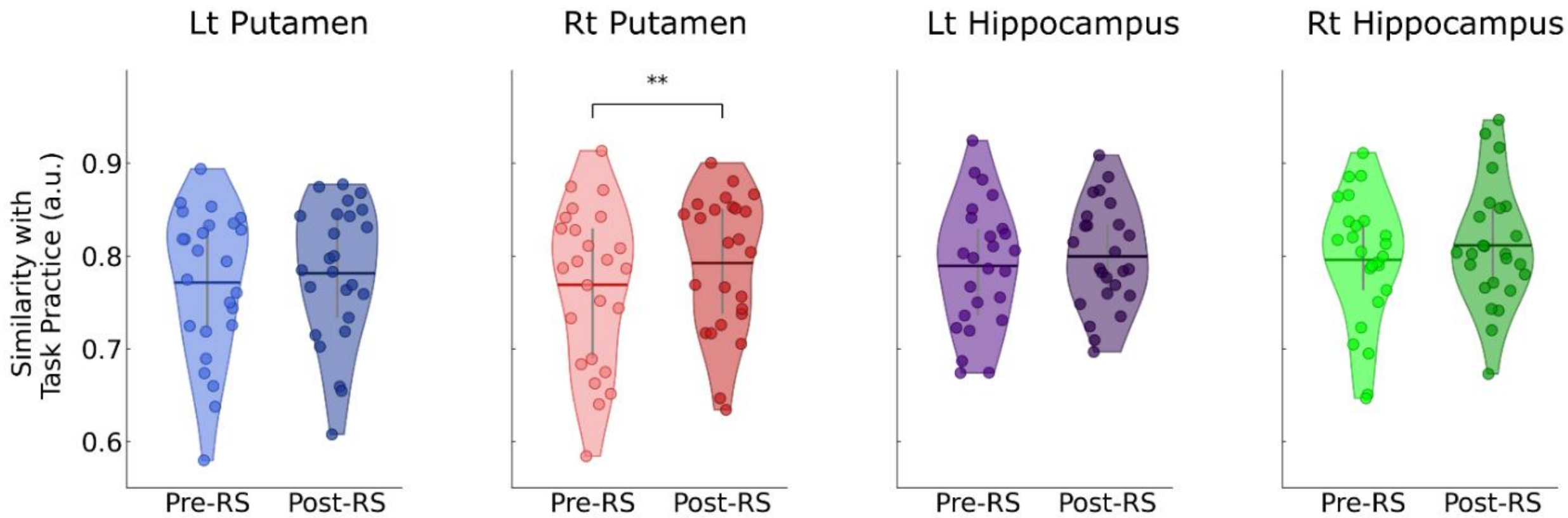

Figure S6. Similarity between task practice and resting state, Related to Figure 5

Violin plots illustrating the representational structure (MVCS) similarity between the neural pattern established during task practice and the patterns observed during pre-learning rest (pre-RS) and post-learning rest (post-RS). Similarity values were computed as Fisher z-transformed Pearson correlation coefficients. Dots within the violin plots represent individual participants' similarity values. The first (25th percentile) and third (75th percentile) quantiles are indicated by the edges of the grey line, and the mean is represented by a solid line. Individual data points are overlaid on the distribution. Statistical significance is indicated by ** ($p < 0.01$, paired two-sided t-test, FDR-corrected for multiple comparisons).

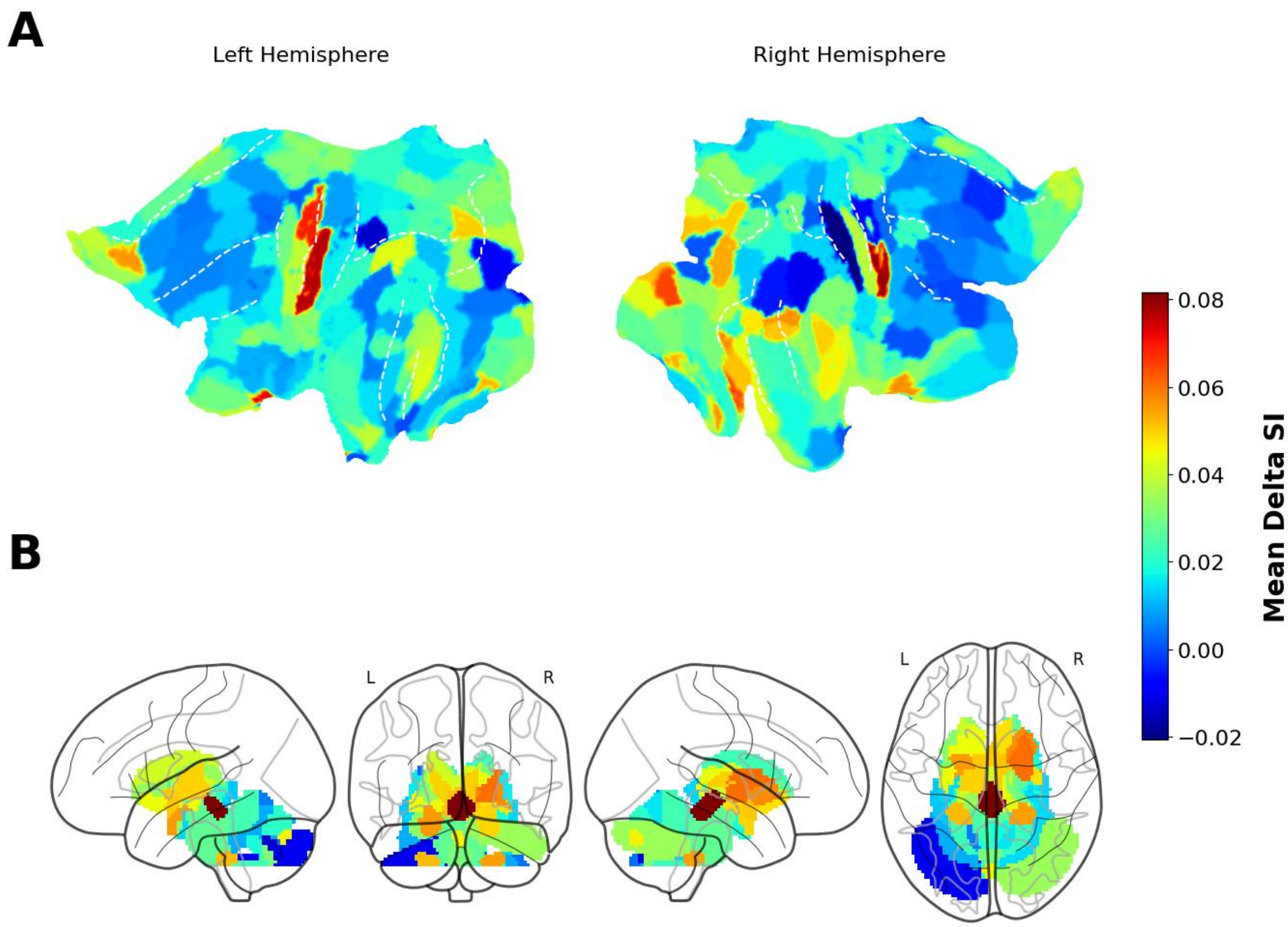

Figure S7. ROI group-level similarity maps, Related to Figure 5

(A) Surface-based visualization of the left and right hemispheres showing the mean delta similarity (SI). White dashed lines indicate major sulcal landmarks. (B) Glass brain view displaying the corresponding subcortical Regions of Interest (ROIs). The color bar indicates the magnitude of the similarity difference, where warmer colors represent higher similarity of the interleaved rest with post-learning rest than pre-learning rest.

A

Fixed effects coefficients	Estimate	Standard Error	z-statistics	P value	Confidence Interval
Intercept	0.623	0.016	39.717	< 0.001	[0.592, 0.654]
Run	0.006	0.002	3.229	0.001	[0.002, 0.009]
Run x Condition	-0.012	0.002	-4.780	< 0.001	[-0.016, -0.007]

B

Fixed effects coefficients	Estimate	Standard Error	z-statistics	P value	Confidence Interval
Intercept	0.643	0.013	47.862	< 0.001	[0.617, 0.670]
Run	0.006	0.002	2.951	0.003	[0.002, 0.009]
Run x Condition	-0.013	0.003	-4.609	< 0.001	[-0.018, -0.007]

C

Fixed effects coefficients	Estimate	Standard Error	z-statistics	P value	Confidence Interval
Intercept	0.618	0.015	41.023	< 0.001	[0.588, 0.647]
Run	0.004	0.002	2.769	0.006	[0.001, 0.007]
Run x Condition	-0.011	0.002	-4.876	< 0.001	[-0.015, -0.006]

D

Fixed effects coefficients	Estimate	Standard Error	z-statistics	P value	Confidence Interval
Intercept	0.632	0.012	51.367	< 0.001	[0.608, 0.656]
Run	0.006	0.002	3.090	0.002	[0.002, 0.010]
Run x Condition	-0.013	0.003	-5.009	< 0.001	[-0.019, -0.008]

Table S1. Table summarizing the mixed-effects model for the similarity with post-RS and pre-RS, Related to Figure 5

Tables summarizing the mixed-effects model result of the similarity between interleaved rest and post/pre-RS as a function of the run for the different ROIs, (A) Right Putamen, (B) Right Hippocampus, (C) Left Putamen and (D) Left Hippocampus.
